# NAC Supplementation of Hyperglycemic Rats Prevents the Development of Insulin Resistance and Improves Antioxidant Status but Only Alleviates General and Salivary Gland Oxidative Stress

**DOI:** 10.1155/2020/8831855

**Published:** 2020-11-14

**Authors:** Anna Zalewska, Sara Zięba, Paula Kostecka-Sochoń, Agnieszka Kossakowska, Małgorzata Żendzian-Piotrowska, Jan Matczuk, Mateusz Maciejczyk

**Affiliations:** ^1^Laboratory of Experimental Dentistry, Medical University of Bialystok, Poland; ^2^Student of the Faculty of Medicine with the Division of Dentistry at the Medical University of Bialystok, Poland; Students Scientific Club “Biochemistry of Civilization Diseases” at the Department of Hygiene, Epidemiology and Ergonomics, Medical University of Bialystok, Poland; ^3^Conservative Dentistry Department, Medical University of Bialystok, Poland; ^4^Department of Hygiene, Epidemiology and Ergonomics, Medical University of Bialystok, Poland; ^5^County Veterinary Inspection, Bialystok, Poland

## Abstract

Previous studies based on animal models demonstrated that N-acetylcysteine (NAC) prevents oxidative stress and improves salivary gland function when the NAC supplementation starts simultaneously with insulin resistance (IR) induction. This study is the first to evaluate the effect of a 4-week NAC supply on the antioxidant barrier and oxidative stress in Wistar rats after six weeks of high-fat diet (HFD) intake. Redox biomarkers were evaluated in the parotid (PG) and submandibular (SMG) salivary glands and stimulated whole saliva (SWS), as well as in the plasma and serum. We demonstrated that the activity of salivary peroxidase and superoxide dismutase and total antioxidant capacity were significantly higher in PG, SMG, and SWS of IR rats treated with NAC. It appears that in PG and SMG of rats fed an HFD, N-acetylcysteine supplementation abolishes oxidative modifications to proteins (evidenced by decreased content of advanced oxidation protein products (AOPP) and advanced glycation end products (AGE)). Simultaneously, it does not reverse oxidative modifications of lipids (as seen in increased concentration of 8-isoprostanes and 4-hydroxynonenal vs. the control), although it reduces the peroxidation of salivary lipids in relation to the group fed a high-fat diet alone. NAC administration increased protein levels in PG and SMG but did not affect saliva secretion, which was significantly lower compared to the controls. To sum up, the inclusion of NAC supplementation after six weeks of HFD feeding was effective in improving the general and salivary gland antioxidant status. Nevertheless, NAC did not eliminate salivary oxidative stress and only partially prevented salivary gland dysfunction.

## 1. Introduction

Insulin resistance (IR) is when the tissues are not sufficiently sensitive to insulin's effects despite its normal or more often elevated blood levels. IR, together with obesity and severe systemic inflammation, play a vital role in the development of the so-called metabolic syndrome and cardiovascular diseases, psychiatric disorders, and cancers [1]. This applies especially to overweight and obese people who are at the highest risk of developing insulin resistance.

Oxidative stress (OS) seems to play a crucial role in the development of IR, which results from chronic inflammation and an intensification of the production of superoxide anion by NADPH oxidase 4 (NOX4), as well as the reduced capacity of antioxidant systems [2–6]. It should be noted that OS is defined as the overproduction of oxygen and nitrogen free radicals exceeding the capacity of the antioxidant barrier responsible for their neutralization. Due to this imbalance, an increase in oxidative modifications of proteins, lipids, DNA, and sugar compounds of the cell is observed, impairing the cells' function and, consequently, the entire organs [7, 8].

Evidence showed that exogenous antioxidants could support or even be used to treat IR and its complications. One of the more important intracellular antioxidants is thiol groups present in proteins and low molecular weight compounds, including glutathione (GSH). Direct GSH supplementation is ineffective as the process of GSH penetration through cell membranes is very negligible. Moreover, mitochondrial membranes are not able to actively transport this molecule at all. Therefore, GSH must be generated intracellularly from endogenous precursors [9]. N-Acetylcysteine (NAC) is one of the precursors [10]. NAC supplementation has a positive effect on GSH reductase (GR), which leads to the elevation of reduced glutathione concentration [11]. Antioxidant mechanisms conditioned by NAC are manifold. It was shown that NAC maintains the disulfide groups of proteins in a reduced state [12], prevents the Fenton reaction [13], indirectly strengthens the enzymatic antioxidant barrier [14], and inhibits peroxynitrite formation [12].

Evidence showed that long-term NAC treatment normalizes glucose metabolism in rats when the supplementation starts parallel to the high-fat diet [2, 6, 14–16]. Similarly, in high-sucrose [17] and high-fat intake conditions [14], NAC supplementation normalizes the lipidemic profile and alleviates hyperglycemia while shifting the redox balance towards the reduction state. NAC offers a significant beneficial effect on sensory neuropathy, cardiovascular disorders, and IR in a similar way to ramipril, which indicates NAC's involvement in preventing IR and associated symptoms [18]. In addition to the direct antioxidant effect, NAC reacts with enzyme increasing their activity through redox regulation [19]. It is believed that NAC supplementation may be beneficial for patients undergoing cardiac surgery [20] and reduce oxidative DNA modifications in children with *β*-thalassemia [21]. NAC is also a potent vasodilator because it can stimulate inducible and endothelial nitric oxide synthase [22–24].

OS, in the course of IR, contributes to the development of many of its complications, including salivary gland dysfunction. In our previous studies, we demonstrated that HFD-induced IR disturbs the antioxidant systems more significantly and entails higher intensity and a greater variety of oxidative modifications in the parotid salivary glands than the submandibular glands [3, 25]. We also observed secretory insufficiency of the parotid glands [3, 25]. NAC supplementation, introduced simultaneously with a high-fat diet, strengthened the antioxidant barrier of both salivary glands, prevented oxidative stress, and restored parotid glands' secretory function. However, we have only observed a reduction in the severity of oxidative modifications in rats' submandibular glands [14].

The results presented above indicate that NAC supplementation effectively prevents salivary gland complications connected with IR induced by a high-fat diet. However, whether NAC can reverse the existing HFD-induced hyperglycemia and its salivary complications remains an open question.

Thus, the study's primary purpose was to assess whether HFD-induced hyperglycemia and oxidative stress of salivary glands and their dysfunction may be abolished by NAC ingestion.

## 2. Materials and Methods

### 2.1. Animals

The Local Committee for Experiments on Animals (Bialystok, Poland) gave a positive opinion and approved the current experiment (No. 21/2017).

The experiment was conducted on sexually mature male Wistar rats (*n* = 40) with an initial body weight of about 69–72 g. Rats were given standard living conditions: air temperature 20–21°C, 12 hours of light/12 hours of darkness, humidity dependent on external environmental conditions, and unlimited access to drinking water. For five days from the moment the rats arrived in the animal research facility, they were fed granulated diet type LSM (Agropol, Motycz, Poland) of the following composition: 10.3% fats, 24.2% proteins, and 65.5% carbohydrates. After five days, the rats were divided into two equal groups as follows:

- Control group (C, *n* = 20): rats receiving standard LSM diet (Agropol, Motycz, Poland) containing 10.3% fats, 24.2% proteins, and 65.5% carbohydrates
- HFD group (*n* = 20): a group of rats fed high-fat diet (Research Diet, USA, catalog number D12492) containing 59.8% fats, 20.1% proteins, and 20.1% carbohydrates [4]

After six weeks, in blood drawn from the tail vein, glucose concentration was determined with a glucometer. In the HFD group, hyperglycemia was confirmed, while in the control rats, the blood glucose level was normal.

After six weeks, rats from every group were randomly divided into two equal subgroups:

- Control group (C, *n* = 10): rats fed standard feed of type LSM (Agropol, Motycz, Poland: 10.3% fats, 24.2% proteins, and 65.5% carbohydrates) as well as a saline solution of 2 mL/kg body weight intragastrically once a day, daily for four weeks
- Group II (C+NAC, *n* = 10): rats fed standard LSM feed as well as NAC solution (500 mg/kg body weight, volume 2 mL/kg body weight (NAC, Sigma A9165)) intragastrically once a day, daily for four weeks [5, 14]
- Group III (HFD, *n* = 10): rats receiving high-fat feed (Research Diet, USA, catalog number D12492: 59.8% fats, 20.1% proteins, and 20.1% carbohydrates) [10], as well as saline solution at a volume of 2 mL/kg body weight intragastrically once a day, every day for four weeks [5, 14]
- Group IV (HFD+NAC, *n* = 10): rats receiving the above-mentioned high-fat feed as well as a solution of NAC (500 mg/kg body weight, the volume of 2 mL/kg body weight (NAC, Sigma, A9165)) intragastrically once a day, every day for four weeks [5, 14]

The NAC dose was determined based on a literature review, which shows that 500 mg/kg body weight is toxicologically safe and sufficient to achieve an antioxidant effect [26]. The NAC dose was selected every two days, after measuring the body weight of rats. A saline and NAC administration took place between 8 and 9 AM. Food intake was monitored twice a week.

### 2.2. Tissue Collection

Rat anesthesia (phenobarbital at a dose 80mg × kg^−1^, intraperitoneally) was preceded by overnight fasting and took place four weeks after introducing NAC supplementation. Once anesthetized rats, the glucose was measured in venous blood collected from the tail vein [25] and the nonstimulated and stimulated (pilocarpine hydrochloride -5 mg/kg body weight, *ip.*; Sigma, Chemical Co., St. Louis, MO, USA) saliva was collected [6]. Cotton rolls containing stimulated saliva were centrifuged in Salivette® tubes, and the obtained material was prepared for further determinations. The amount of the collected unstimulated saliva was insufficient for any analyses, while the quantity of stimulated saliva was adequate to determine the antioxidant activity/concentration.

Blood was drawn from the abdominal artery and centrifuged to obtain plasma. The plasma was stored at -80°C [3].

The submandibular and parotid salivary glands of rats were collected, weighed, frozen in liquid nitrogen, and stored at -80°C.

Two weeks after, the salivary glands have been prepared for biochemical determinations: thawed, homogenized in PBS (1 : 10, protease inhibitors -1 tablet/10 mL buffer) (Omni TH, Omni International, Kennesaw, GA, USA), sonified (1800 J/20 sec, three times, on ice, UP400S, Hielscher, Teltow, Germany), and centrifuged (20 minutes, 4°C, 5000 g, MPW, Med. Instruments, Warsaw, Poland). The supernatant fluid was preserved for further assays [3].

BHT (Sigma-Aldrich, Germany) was added to serum and PBS (10 *μ*L 0.5 M BHT/1 mL).

The colorimetric method (Thermo Scientific Pierce BCA Protein Assay Kit, Rockford, IL, USA; wavelength: 562 nm) was used to assess the protein concentration. Bovine serum albumin (BSA) was used as a standard.

### 2.3. Salivary/Serum Enzymatic Antioxidants

We followed the methods of Choromańska et al. [27] and Zalewska et al. [5].

To determine salivary/serum superoxide dismutase (SOD, E.C. 1.15.1.1) activity, the spectrophotometric method developed by Misra and Fridovich [28] was used. Adrenaline oxidation to adrenochrome was measured at wavelength 480 nm. One unit of SOD activity in saliva/serum was expressed as the amount of enzyme that reduces adrenaline oxidation by 50%.

To determine salivary/serum catalase (CAT, E.C. 1.11.1.6) activity, the spectrophotometric method developed by Aebi [29] was used. This method evaluates the rate of hydrogen peroxide decomposition in the sample, which is measured at the wavelength of 240 nm. One unit of salivary/serum CAT activity was expressed as the amount of the enzyme which breaks down one nmol of hydrogen peroxide in the sample in 1 minute [29].

The method of Mansson-Rahemtulla et al. [30] was used to determine the salivary peroxidase (Px, E.C. 1.11.1.7) activity. Briefly, thionitrobenzoic acid is produced by reducing 5,5′-dithiobis-(2-nitrobenzoic acid) (DTNB). Thionitrobenzoic acid reacts with thiocyanate anions (SCN-) produced as a result of the oxidation reaction of potassium thiocyanate (KSCN) by Px. The absorbance was measured at 412 nm.

Paglia and Valentine's [31] method was used to assess serum glutathione peroxidase (GPx, E.C. 1.11.1.9). This method involves the conversion of NADPH (reduced nicotinamide adenine dinucleotide phosphate) to NADP^+^ (reduced nicotinamide adenine dinucleotide phosphate). The measurements were performed at 340 nm. One unit of serum GPx activity was defined as the enzyme's amount to catalyze the oxidation of 1 *μ*mol of NADPH for 1 minute.

Salivary/serum glutathione reductase (GR, E.C. 1.8.1.7) activity was determined spectrophotometrically at a wavelength of 340 nm, according to the method by Mize and Langdon [32]. One unit of this enzyme activity was represented as the amount of enzyme necessary to carry out the oxidation reaction of 1 *μ*mol of NADPH per 1 minute.

### 2.4. Salivary/Plasma Nonenzymatic Antioxidants

Salivary/plasma uric acid (UA) concentration was determined by the colorimetric method using the reagent kit QuantiChrom TM Uric Acid Assay Kit DIUA-250 (BioAssay Systems, Hayward, CA, USA) according to manufacturer's instruction.

Salivary/plasma ascorbic acid (AA) concentration was determined colorimetrically using the Folin and Ciocalteu phenol reagent. The method is based on the reduction of the Folin reagent under the influence of AA. The measurement was taken at 760 nm wavelength [33].

The concentration of salivary/plasma reduced glutathione (GSH) was determined colorimetrically. The reduction of DTNB to 2-nitro-5-mercaptobenzoic acid under the influence of GSH was examined. The absorbance of the samples was measured at 412 nm wavelength [34].

### 2.5. Salivary/Plasma Redox Status

Salivary/plasma total antioxidant capacity (TAC) was determined at a 660 nm wavelength, by the colorimetric method using 2,2′-azino-bis-3-ethylbenzothiazoline-6-sulfonic acid radical cation (ABTS^∗^^+^) [35]. This method is based on changes in the absorbance of the ABTS^∗^^+^ solution caused by antioxidants contained in the analyzed sample.

Salivary/plasma total oxidant status (TOS) was measured colorimetrically as described by Erel [36]. This method uses the oxidation reaction of Fe^2+^ to Fe^3+^ ions, which occurs in the presence of oxidants in the biological material. Detection of Fe^3+^ ions enabled the use of xylenol orange (XO).

Oxidative stress index (OSI) was calculated using the formula: TOS/TAC × 100% [37].

### 2.6. Salivary/Plasma Oxidative Stress

Kalousová et al.'s [38] method was used to assess the concentration of salivary/plasma advanced oxidation protein products (AOPP) and advanced glycation end products (AGE). Before making determinations, biological materials (saliva and plasma) were diluted (1 : 5, *v*/*v*) with PBS. For AOPP measurement, changes in the absorbance caused by the iodine ion's oxidative capacity were measured at 340 nm. AGE-specific fluorescence at 350 nm excitation wavelength and 440 nm emission wavelength was measured [38].

Salivary/plasma 8-isoprostane (8-isoP) concentration was measured with the ELISA method using the entire set of reagents (8-Isoprostane ELISA Kit, Cayman Chemical, Ann Arbor, MI, USA). This method is based on the competition between 8-isoP and 8-isoP conjugate with acetylcholinesterase (Tracer). The concentration of 8-isoP in the test sample is inversely proportional to the binding capacity of the 8-isoP conjugate to acetylcholinesterase and the antibody.

Salivary/plasma 4-HNE-protein adduct (4-HNE) concentration was assessed by ELISA using commercial sets (Cell Biolabs, Inc., San Diego, CA, USA; USCN Life Science, Wuhan, China) following the manufacturer's instructions included in the package.

Salivary/plasma 8-hydroxy-D-guanosine (8-OHdG) concentration was measured by the ELISA method using a commercial kit (USCN Life Science, Wuhan, China) according to the recommendations of its producer. This test is based on the reactions between a specific fragment of 8-OHdG protein chain with appropriate monoclonal antibodies and reagents that eventually cause a colored reaction. The color intensity is proportional to the concentration of 8-OHdG in the test sample and is measured at 450 nm wavelength.

### 2.7. Statistics

The obtained results were statistically analyzed using GraphPad Prism 8 for MacOS (GraphPad Software, La Jolla, CA, USA). The Shapiro-Wilk and Kolmogorov-Smirnov tests were used to check the distribution of variables. We also used Levene's test, which is aimed at analyzing the uniformity of variance. Due to the normal distribution of results, the ANOVA test was used with Tukey's HSD test for multiple comparisons. Multiplicity-adjusted *p* value was also calculated. Moreover, Pearson's linear correlation coefficient was used between the obtained results. We assumed the value of *p* < 0.05 as statistically significant.

## 3. Results

### 3.1. Before NAC Supplementation

The food intake of rats fed HFD was significantly lower (-29%, *p* ≤ 0.0001), and their body weight was significantly elevated compared to the control group (+18%, *p* ≤ 0.0001). The concentration of tail blood glucose after six weeks of high-fat diet consumption was significantly higher compared to the control (+39%, *p* ≤ 0.0001) (Table 1).

### 3.2. After NAC Supplementation

Similar to the period before supplementation, although feed intake of rats on a high-fat diet was significantly lower (-40%, *p* ≤ 0.0001), their body weight was considerably higher than the control group (+28%, *p* ≤ 0.0001). In the rats fed an HFD and supplemented with NAC, feed intake was significantly higher (+23%, *p* = 0.0733), and body weight was significantly lower than the HFD group (-19%, *p* ≤ 0.0001). Glucose, insulin, and HOMA-IR levels in the HFD group were significantly higher compared to the controls (+39%, *p* ≤ 0.0001; +55%, *p* ≤ 0.0001; and +72%, *p* ≤ 0.0001, respectively) and the NAC-supplemented group (+27%, *p* ≤ 0.0001; +41%, *p* ≤ 0.0001; and +57%, *p* ≤ 0.0001, respectively). We did not observe any differences in the evaluated parameters between the groups C and C+NAC or C and HFD+NAC (Table 1).

The weight of PG and SMG of HFD rats was significantly higher than in the controls (+25%, *p* ≤ 0.0001 and +22%, *p* ≤ 0.0001, respectively); only the weight of the submandibular gland decreased after introducing NAC supplementation (-23%, *p* = 0.0002) (Table 2).

Total protein content in the homogenate of the parotid and submandibular salivary glands of rats fed a high-fat diet was significantly decreased vs. control (-36%, *p* ≤ 0.0001 and -28%, *p* ≤ 0.0001, respectively). After NAC supplementation, total protein content in the homogenate of the parotid and submandibular salivary glands of HFD rat diet was considerably higher compared to total protein content in the homogenates of these salivary glands in HFD rodents (+39%, *p* ≤ 0.0001 and +20%, *p* = 0.01, respectively), and it reached the control group level. Stimulated saliva secretion in HFD rats was significantly lower than in the control group (-28%, *p* = 0.02). NAC administration did not affect saliva's stimulated secretion, resulting in considerably lower secretion in HFD+NAC rats than the controls (-21%, *p* = 0.02). Unstimulated secretion did not differ between the groups (Table 2).

### 3.3. Plasma

High-fat diet resulted in a significant decrease in the plasma activity of SOD and CAT in HFD rats compared to the control group (-61%, *p* ≤ 0.0001 and -53%, *p* ≤ 0.0001, respectively). NAC supplementation commenced after 6 weeks of HFD intake significantly boosted SOD and CAT activities in the HFD+NAC group in comparison with the HFD group (+38%, *p* = 0.001 and +30%, *p* ≤ 0.0001, respectively), reaching the level observed in the control group. Similarly, a high-fat diet decreased plasma concentrations of AA, GSH, and TAC of HFD rats compared to the control (-19%, *p* = 0.0007; -61%, *p* ≤ 0.0001; and -52%, *p* ≤ 0.0001, respectively). NAC supplementation for 4 weeks elevated plasma levels of AA, GSH, and TAC of rats from the HFD+NAC group compared to the HFD group (+15%, *p* = 0.02; +59%, *p* ≤ 0.0001; and +36%, *p* = 0.006, respectively). The concentrations of UA, TOS, OSI, 8-isoP, 4-HNE, AGE, AOPP, and 8-OHdG were considerably higher in the plasma of rats fed a high-fat diet compared to the control group (+31%, *p* = 0.006; +63%, *p* ≤ 0.0001; +83%, *p* ≤ 0.0001; +54%, *p* = 0.0002; +58%, *p* ≤ 0.0001; +50%, *p* ≤ 0.0001; +40%, *p* = 0.0001; and +60%, *p* = 0.0006, respectively). Four-week NAC supplementation reduced OSI as well as 4-HNE concentration in HFD+NAC rats compared to the group only fed a high-fat diet (-42%, *p* = 0.0004 and -17%, *p* = 0.007, respectively), with these values being significantly higher than in the control group (+70%, *p* = 0.0008 and +50%, *p* ≤ 0.0001, respectively). Despite NAC supplementation in the HFD+NAC group, TOS values were considerably elevated compared to the control group (+58%, *p* ≤ 0.0001). In the group of rats fed a high-fat diet and supplemented with NAC, 8-isoP and AGE plasma concentrations were not decreased compared to the HFD group, and only the plasma level of 8-isoP was significantly increased compared to the control group (+40%, *p* = 0.04). Plasma concentration of AOPP in HFD+NAC rats was decreased in comparison with the HFD group (-30%, *p* = 0.002) to the level observed in the control group (Table 3).

### 3.4. Enzymatic Antioxidants

#### 3.4.1. Parotid Salivary Glands

**Figure 1:**
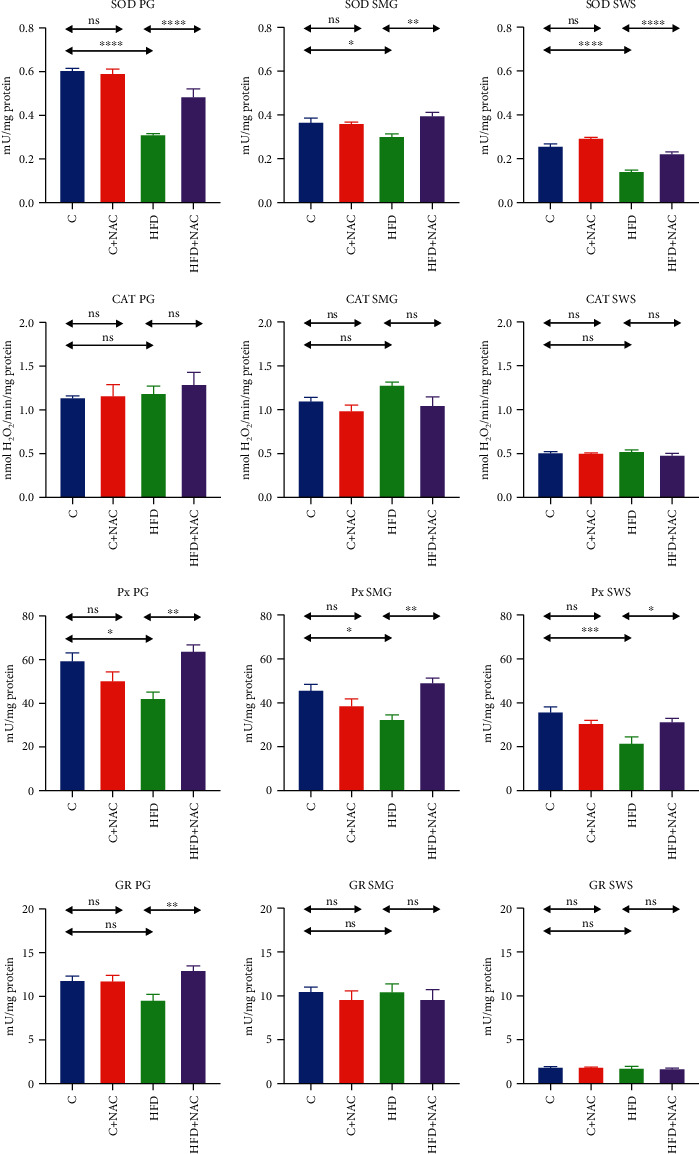
Effect of N-acetylcysteine on salivary antioxidant enzymes of rats. C: control group; HFD: high-fat-fed group; C+NAC: control rats+N-acetylcysteine supplementation; HFD+NAC: high-fat-fed rats+N-acetylcysteine supplementation; SOD: superoxide dismutase; CAT: catalase; Px: salivary peroxidase; GR: glutathione reductase; ^∗^*p* < 0.05, ^∗∗^*p* < 0.005, ^∗∗∗^*p* < 0.0005, and ^∗∗∗∗^*p* < 0.0001.

SOD and Px's activities in the homogenate of parotid salivary glands of HFD rats were significantly lower than in the control group (-48%, *p* ≤ 0.0001 and -29%, *p* = 0.01, respectively). NAC supplementation considerably increased the activity of SOD and Px in the group fed a high-fat diet (HFD+NAC) compared to the HFD group without supplementation (+36%, *p* ≤ 0.0001 and +34%, *p* = 0.001, respectively), to the level observed in the control group (Figure 1).

CAT activity in the parotid glands' homogenates did not differ significantly between the studied groups (Figure 1).

Although a high-fat diet did not considerably decrease GR activity compared to the control group, NAC supplementation boosted GR activity in the group fed a high-fat diet (HFD+NAC) in comparison with the HFD group (+26%, *p* = 0.004), elevating it to the level observed in the control group (Figure 1).

#### 3.4.2. Submandibular Salivary Glands

The activities of SOD and Px in the homogenate of submandibular salivary glands of HFD rats were significantly lower than in the control group (-18%, *p* = 0.04 and -29%, *p* = 0.01, respectively). NAC supplementation significantly increased the activities of SOD and Px in the group fed a high-fat diet (HFD+NAC) compared to the HFD group without supplementation (+23%, *p* = 0.002 and +34%, *p* = 0.001, respectively), reaching the level noted in the control group (Figure 1).

#### 3.4.3. Stimulated Whole Saliva (SWS)

SOD and Px activities in stimulated saliva of HFD rats were significantly lower than in the control group (-44%, *p* ≤ 0.0001 and -40%, *p* = 0.0009, respectively). NAC supplementation considerably boosted SOD and Px activities in stimulated saliva of HFD+NAC rats compared to the HFD group without supplementation (+36%, *p* ≤ 0.0001 and +31%, *p* = 0.03, respectively), to the level observed in the control group (Figure 1).

### 3.5. Nonenzymatic Antioxidants

#### 3.5.1. Parotid Salivary Glands

**Figure 2:**
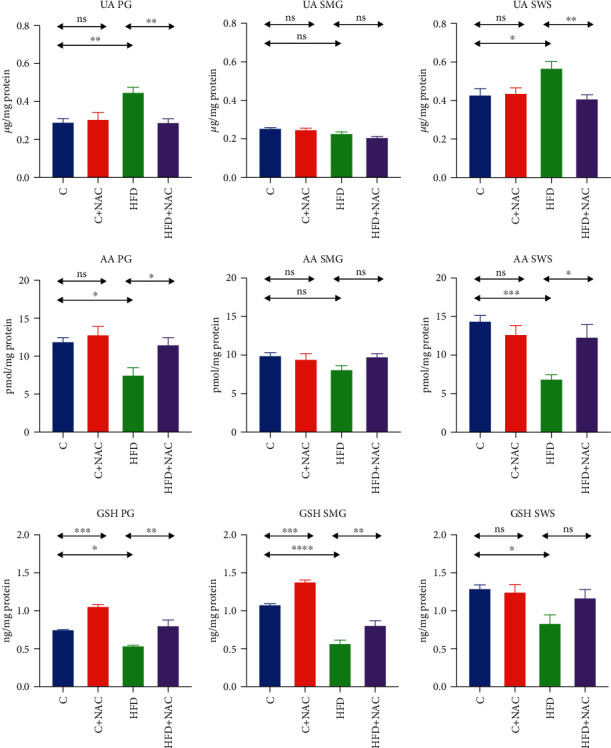
Effect of N-acetylcysteine on salivary nonenzymatic antioxidants of rats. C: control group; HFD: high-fat-fed group; C+NAC: control rats+N-acetylcysteine supplementation; HFD+NAC: high-fat-fed rats+N-acetylcysteine supplementation; UA: uric acid; AA: ascorbic acid; GSH: reduced glutathione; ^∗^*p* < 0.05, ^∗∗^*p* < 0.005, and ^∗∗∗^*p* < 0.0005.

The concentration of UA in the homogenate of parotid salivary glands of rats fed a high-fat diet was significantly higher than in the control group (+35%, *p* = 0.003). NAC supplementation considerably lowered UA concentration in the HFD+NAC group compared to the HFD group (-35%, *p* = 0.003), reaching the level comparable to that in the control group (Figure 2).

AA concentration in the homogenate of the parotid salivary glands of rats fed a high-fat diet was significantly lower than in group C (-37%, *p* = 0.02). NAC supplementation considerably elevated AA concentration in the HFD+NAC group compared to the HFD group (+35%, *p* = 0.03) to the level observed in the control group (Figure 2).

The concentration of GSH in the homogenate of the parotid salivary glands of HFD rats was significantly lower than in the control group (-28%, *p* = 0.01). NAC supplementation caused a considerable increase in GSH concentration in the group fed a high-fat diet (HFD+NAC) compared to the HFD group without supplementation (+33%, *p* = 0.001), reaching the level observed in the control group. Interestingly, NAC supplementation raised the concentration of GSH in the C+NAC group compared to the control group (+29%, *p* = 0.0002) (Figure 2).

#### 3.5.2. Submandibular Salivary Glands

The concentration of GSH in the homogenate of the submandibular salivary glands of HFD rats was significantly lower than in the control group (-47%, *p* ≤ 0.0001). NAC supplementation notably boosted GSH concentration in the group fed a high-fat diet (HFD+NAC) compared to the HFD group without supplementation (+29%, *p* = 0.005) to respective level observed in the control group. Similar to the parotid salivary glands, NAC supplementation increased GSH concentration in the C+NAC group compared to the control group (+22%, *p* = 0.0004) (Figure 2).

#### 3.5.3. Stimulated Saliva

UA's concentration in the stimulated saliva of rats fed a high-fat diet was significantly higher than in the control group (+24%, *p* = 0.03). NAC supplementation considerably lowered UA concentration in the HFD+NAC group compared to the HFD group (-28%, *p* = 0.009), to the level observed in the control group (Figure 2).

AA concentration in the stimulated saliva of HFD rats was significantly lower than in the controls (-52%, *p* = 0.0004). NAC supplementation significantly increased AA concentration in the HFD+NAC group compared to HFD rats (+44%, *p* = 0.01), to the level found in the control group (Figure 2).

The concentration of GSH in the stimulated saliva of HFD rats was significantly lower than in the controls (-35%, *p* = 0.02). NAC supplementation did not notably affect GSH concentration in HFD+NAC rats' saliva than the controls and the HFD group (Figure 2).

### 3.6. Redox Status

#### 3.6.1. Parotid Salivary Glands

**Figure 3:**
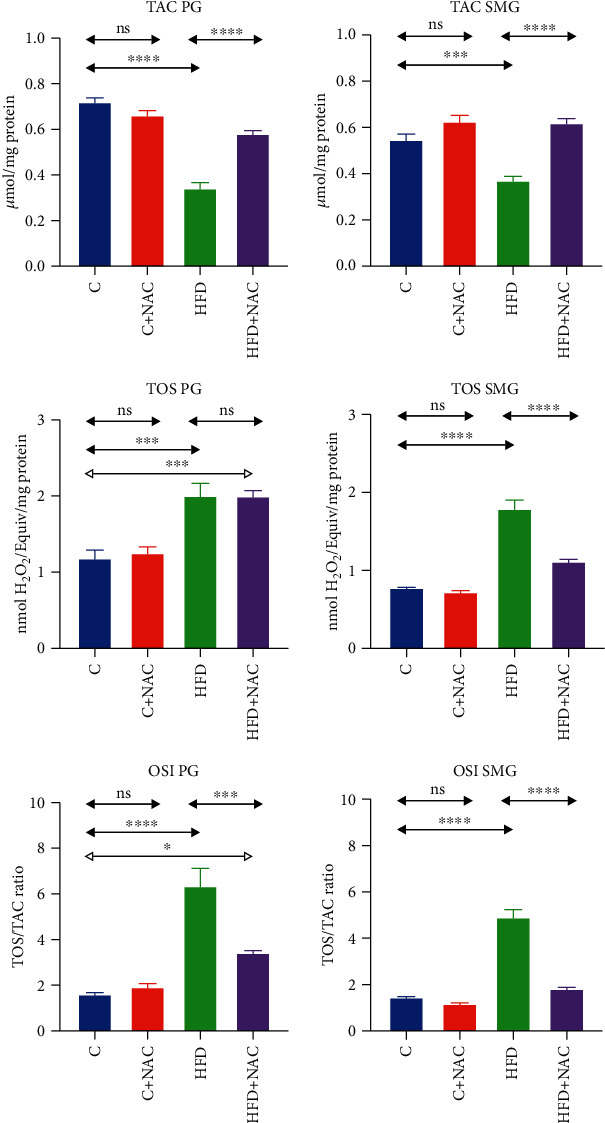
Effect of N-acetylcysteine on the salivary redox status of rats. C: control group; HFD: high-fat-fed group; C+NAC: control rats+N-acetylcysteine supplementation; HFD+NAC: high-fat-fed rats+N-acetylcysteine supplementation; TAC: total antioxidant capacity; TOS: total oxidant status; OSI: oxidative status index; ^∗^*p* < 0.05, ^∗∗∗^*p* < 0.0005, and ^∗∗∗∗^*p* < 0.0001.

TAC in the homogenate of the parotid glands of HFD rats was significantly lower than the control group (-52%, *p* ≤ 0.0001). NAC supplementation boosted TAC in the homogenate of the parotid glands in HFD+NAC rats compared to the HFD group (+41%, *p* ≤ 0.0001), to the levels observed in the control group (Figure 3).

TOS and OSI in the homogenate of the parotid salivary glands of HFD rats were significantly higher compared to the controls (+41%, *p* = 0.0003 and +70%, *p* ≤ 0.0001, respectively), while NAC supplementation decreased OSI in the parotid gland homogenate of HFD+NAC rats compared to the HFD group (-46%, *p* ≤ 0.0001, respectively). Despite NAC supplementation, both TOS and OSI values in the parotid salivary glands of HFD+NAC rats were significantly higher compared to the control group (+49%, *p* = 0.0005 and 53%, *p* = 0.03, respectively) (Figure 3).

#### 3.6.2. Submandibular Salivary Glands

TAC in the homogenate of the submandibular salivary glands of HFD rats was significantly lower than the control group (-32%, *p* = 0.0005). The 4-week NAC supplementation increased TAC in the submandibular salivary gland homogenate of HFD+NAC rats compared to the HFD group (+40%, *p* ≤ 0.0001) to the levels observed in the control group (Figure 3).

TOS and OSI in the homogenate of the submandibular salivary glands of HFD rats were considerably higher than the control group (+56%, *p* ≤ 0.0001 and +70%, *p* ≤ 0.0001, respectively). Four-week NAC supplementation decreased TOS and OSI in the homogenate of the submandibular salivary glands of HFD+NAC rats compared to the HFD group (-38%, *p* ≤ 0.0001 and -63%, *p* ≤ 0.0001, respectively), to the levels observed in the control rats (Figure 3).

### 3.7. Oxidation Products

#### 3.7.1. Parotid Salivary Glands

**Figure 4:**
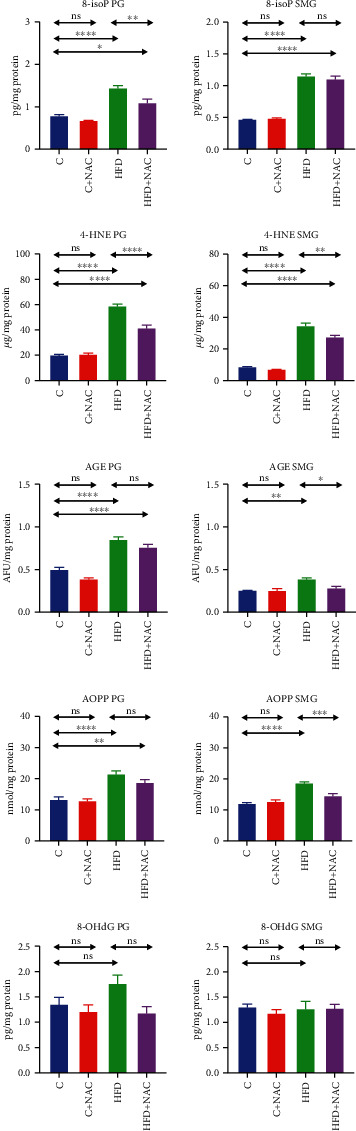
Effect of N-acetylcysteine on oxidative damage to salivary proteins, lipids, and DNA of rats. C: control group; HFD: high-fat-fed group; C+NAC: control rats+N-acetylcysteine supplementation; HFD+NAC: high-fat-fed rats+N-acetylcysteine supplementation; 8-isoP: 8-isoprostanes; 4-HNE: 4 hydroxynonneal protein adducts; AGE: advanced glycation end products; AOPP: advanced oxidation protein products; 8-OHdG: 8-hydroxy-d-guanosine; ^∗^*p* < 0.05, ^∗∗^*p* < 0.005, ^∗∗∗^*p* < 0.0005, and ^∗∗∗∗^*p* < 0.0001.

High-fat diet increased the concentrations of 8-isoP, 4-HNE, AGE, and AOPP in the homogenate of the parotid salivary glands of HFD rats compared to the control (+45%, *p* ≤ 0.0001; +66%, *p* ≤ 0.0001; +41%, *p* ≤ 0.0001; and +38%, *p* ≤ 0.0001, respectively). Four-week NAC supplementation decreased the concentrations of 8-isoP and 4-HNE in the parotid glands of HFD+NAC rats compared to the HFD group (-25%, *p* = 0.003 and -30%, *p* = 0.0001, respectively), while they remained significantly higher compared to the control group (+28%, *p* = 0.01 and +52%, *p* = 0.0001, respectively). Despite NAC supplementation, AGE and AOPP concentrations in HFD+NAC rats did not differ from those observed in the HFD group and were considerably higher compared to the controls (+36%, *p* = 0.0001 and +30%, *p* = 0.0001, respectively) (Figure 4).

#### 3.7.2. Submandibular Salivary Glands

High-fat diet caused a significant increase in the concentrations of 8-isoP, 4-HNE, AGE, and AOPP in the submandibular salivary gland homogenate of HFD rats compared to the control (+59%, *p* ≤ 0.0001; +75%, *p* ≤ 0.0001; +34%, *p* = 0.001; and +36%, *p* ≤ 0.0001, respectively). Four-week NAC supplementation lowered the concentrations of AGE and AOPP to the levels observed in the control group, while in the HFD+NAC group, these values were significantly decreased compared to the HFD group (-27%, *p* = 0.01 and -22%, *p* = 0.0009, respectively). Despite NAC supplementation, the concentration of 8-isoP in HFD+NAC rats did not differ from that observed in the HFD group and was considerably higher compared to the control group (+58%, *p* = 0.0001). As a result of NAC supplementation, 4-HNE content in the HFD+NAC group was significantly lower than in rats fed exclusively an HFD (-21%, *p* = 0.002), although it remained notably higher compared to the control group (+79%, *p* = 0.0001) (Figure 4).

### 3.8. Correlations

We demonstrated a positive correlation between TAC and GSH in both salivary glands of HFD+NAC rats (PG: *r* = 0.650, *p* = 0.042; SMG: *r* = 0.812, *p* = 0.009). There was also a positive relationship between UA and TOS concentrations in the parotid salivary glands (*r* = 0.836, *p* = 0.003).

We observed a negative correlation between 8-isoP concentration in the parotid glands and the rate of stimulated saliva secretion in the HFD+NAC group (*r* = −0.763, *p* = 0.001). Moreover, a positive correlation was noted between HOMA-IR index and protein concentration in both salivary glands (PG: *r* = 0.693, *p* = 0.026; SMG: *r* = 0.787, *p* = 0.007).

## 4. Discussion

IR is a condition that leads to numerous systemic diseases of devastating impact. Therefore, it is crucial to counteract its development and prevent deepening the existing pathologies in this metabolic disorder.

In the presented experiment, we were the first to demonstrate the beneficial effect of NAC supplementation in strengthening antioxidant defense and reversing OS symptoms and salivary gland dysfunction in rats with hyperglycemia induced by a high-fat diet. Moreover, the inclusion of NAC supplementation at the hyperglycemia stage induced by a 6-week high-fat diet eliminated hyperglycemia and, more importantly, prevented IR development.

According to our previous research, only five weeks of HFD is enough to develop IR and general and salivary gland OS [25]. However, in the model presented herein, we could only control the blood glucose level after six weeks. All HFD rats developed a blood glucose level above 150 mg/dL, which confirmed hyperglycemia. HFD-induced IR could only be confirmed after ten weeks of the experiment. Rats from the group fed a high-fat diet exclusively have developed all IR laboratory symptoms: elevated blood glucose and insulin levels and an increase in HOMA-IR compared to the control group. NAC reduces the intestinal absorption of nutrients [17], which most likely results in a decrease in the body weight of rats fed a high-fat diet and supplemented with NAC compared to the HFD group, and also has a positive effect on the studied aspects of carbohydrate metabolism. After six weeks of exposure to HFD, 4-week NAC supplementation lead to normalization of glucose levels, resulting in normal insulin and HOMA-IR levels observed in the control group. It was shown that a decreased insulin level in HFD+NAC-treated rats is most likely due to increased tissue sensitivity to insulin [15].

Our results showed that NAC positively affects eliminating general OS and upregulates antioxidant enzymes and nonenzymatic antioxidants in rats with induced hyperglycemia. A similarly beneficial NAC effect on overall redox balance has been observed in streptozotocin-induced diabetes [23, 24, 39–41]. However, it should be stressed that the administration of NAC boosts the activity/concentration of all antioxidants reduced by the high-fat diet in comparison with the HFD group, bringing it to the levels observed in the plasma of control rats. Simultaneously, the redox balance remains frequently inclined towards oxidation reactions, which means that free radicals' production exceeds the body's ability to neutralize them. Despite NAC supplementation, TOS and OSI values and 4-HNE, 8-isoP, and AGE concentrations in the plasma of the HFD+NAC rats are higher than the control group, even though they remain decreased compared to the HFD group.

Our study confirmed a high-fat diet's adverse effect on redox balance and salivary glands' function, consistent with previous reports [3, 14, 25].

Interestingly, after NAC's introduction, the salivary gland redox balance changes tend to mimic this balance observed in the blood. The inclusion of NAC supplementation at the stage of hyperglycemia strengthens the antioxidant barrier to such an extent that the enzymes' activity and concentration of nonenzymatic antioxidants in both salivary glands after four weeks of supplementation are higher than in rats fed a high-fat diet exclusively. More importantly, the antioxidant defense parameters, including TAC, are at the control group's levels, which indicates their full “recovery” even though rats are still fed an HFD. TAC is one of the essential antioxidant defense parameters because it reflects all the body's antioxidants. A positive correlation between TAC and GSH in both salivary glands of HFD+NAC rats suggests that NAC restores the antioxidant barrier mainly through GSH increase. It seems that NAC supplementation decreases OS in salivary gland cells because, as studies have shown, in mild OS conditions, an increase in glutamate-cysteine ligase activity was observed, which translated into an increase in GSH concentration [42]. Under severe OS conditions, glutathione is used in several processes: oxidation, conjugation, and extrusion from the cell [42]. The phenomenon of NAC-dependent NF-*κ*B suppression that unblocks the antioxidant enzyme genes may explain the increase in SOD and Px activity in PG, SMG, and SWS and GR in PG [43, 44].

The increased concentration of nitrogen-free radicals and their derivatives in salivary glands in the course of HFD-induced IR observed in earlier studies [6, 45] could be one reason for decreased AA level in both salivary glands of HFD rats. It was documented that AA readily reacts with nitrite, especially in increased nitrosative stress [46]. These reactions lead to the production of ascorbate radicals and then dehydroascorbate (DHA). DHA can be reduced back to ascorbate by a GSH-dependent reaction catalyzed by DHA reductase. In the case of GSH deficiency and its excessive consumption in eliminating nitrogen radicals, AA is not sufficiently regenerated, which leads to its deficiency [47]. The NAC administration significantly regenerates the GSH pool, which may be why there is an observed considerable increase in AA concentration in the salivary glands of HFD+NAC rats. Moreover, NAC acts as a free radical scavenger, thus being an additional source of antioxidant potential, it may “lighten” and reduce the use of endogenous antioxidants in free radical control.

Uric acid, the final metabolic product of purine bases, has been considered for years as one of the primary salivary antioxidants. However, its negative correlation with stimulated secretion rate suggests that in the case of HFD nutrition, UA shifts the salivary redox balance towards the oxidation reaction and OS. After NAC supplementation, we observed a significant decrease in UA concentration (in the parotid glands and stimulated saliva) compared to the HFD group, reaching the level noted in the control group. Moreover, we observed a positive correlation between UA and TOS concentrations in the parotid salivary glands, indicating that aminocarbonyl radicals formed with UA's participation are significant sources of free radicals in the parotid glands of rats fed a high-fat diet, despite NAC supplementation [47]. It is worth mentioning that although we did not directly assess the rate of ROS production, we assayed the level of TOS. TOS reflects the content of all oxidants in the sample [48]. The increase in TOS concentration in both salivary glands of HFD rats is, therefore, not surprising. However, the applied NAC supplementation reduced free radicals' concentration to the control group level only in the submandibular glands. In the parotid glands, the TOS level did not change in respect of the HFD group and was significantly higher than in the control group. Another redox equilibrium indicator, OSI, behaves similarly, suggesting the persistent intensification of oxidative reactions in the parotid glands of rats exposed to a high-fat diet with NAC supplementation.

The increase in the intensity of oxidative damage in the parotid glands compared to the rats' submandibular glands confirms the earlier reports [3]. In the submandibular glands, NAC supplementation eliminates oxidative modifications of carbohydrates and proteins (AGE and AOPP contents did not differ from those observed in the control group); however, it does not reverse oxidative modifications of lipids (concentrations of 8-isoP and 4-HNE protein adduct were higher than in the control group). For 4-HNE-protein adducts after NAC supplementation, we observed a 21% decrease in their concentration compared to the HFD group, but it was still 79% higher than in the control group. In the parotid glands, NAC supplementation reduced 8-isoP and 4-HNE-protein adduct levels by 25% and 30%, respectively, compared to the HFD group, but—as in the submandibular glands—these values remained significantly higher compared to the controls (by 28% and 52%, respectively). However, NAC supplementation in the group fed a high-fat diet did not affect AGE and AOPP concentrations in the parotid salivary glands. They remained at a similar level to the values observed in the HFD group and were 36% and 30% higher, respectively, compared to the control group. Based on the presented results, it can be concluded that NAC supplementation applied at the stage of developed hyperglycemia reduces oxidative stress to a greater extent in the submandibular salivary glands versus the parotid glands of rats exposed to a high-fat diet. In addition, the selective presence of lipid peroxidation products is believed to indicate low levels of OS. Evidence has shown that one of the first manifestations of mild oxidative stress is the increase in lipid peroxidation products. This is due to the fact that being highly bioavailable lipids are peroxidized even at low ROS concentrations. In a situation of intensification of oxidative reactions and OS, oxidative modifications of proteins, carbohydrates, and later DNA occur [48].

A high-fat diet did not affect the secretion of unstimulated saliva; however, it led to decreased stimulated saliva secretion, which is consistent with the results of Kołodziej et al. [3]. The changes in stimulated saliva secretion induced by HFD appear to be irreversible as NAC supplementation, with an already developed hyperglycemia stage, did not result in normalization of stimulated saliva secretion. It is believed that stimulated secretion mainly occurs in the parotid salivary glands, which—in our study—seems to confirm a similar pattern of changes in concentration/activities of antioxidants in stimulated saliva and the parotid salivary glands during HFD and NAC intake. Abnormal secretion of stimulated saliva in the course of a high-fat diet is attributed to fatty degeneration of the parenchyma of these salivary glands, neurotransmission alternations, and decreased sensitivity of muscarinic receptors [3, 14, 25, 45]. Given that NAC has an anti-inflammatory and antiapoptotic effect and positive effects on nerve signal transduction [39], it seems unlikely that lowered SWS flow that persists despite NAC supplementation is the result of disturbances at the neurotransmission level or apoptosis. The negative correlation between the concentration of 8-isoP in the parotid glands and the rate of stimulated saliva secretion in the HFD+NAC group may also be significant. Isoprostanes are a potential factor impairing the mucous membrane's integrity and liquidity and the function of membrane receptors [40]. Moreover, isoprostanes have been demonstrated to reduce the release of neurotransmitters by some cells of the human body [49, 50], which would be of the utmost importance in saliva secretion.

NAC supplementation has a positive effect on the synthesis/secretion of protein in the salivary glands. In our study, protein concentration was significantly elevated in both salivary glands compared to the HFD group, reaching the level observed in the control group. A positive correlation between HOMA-IR index and protein concentration in both salivary glands of HFD+NAC rats suggests that increased cell sensitivity to insulin is an essential factor in normalizing protein production in salivary glands.

We would like to emphasize that the current model assesses the redox balance of the salivary glands of HFD-fed rats that are supplemented with NAC introduced during the hyperglycemic stage. Interestingly, NAC supplemented simultaneously with the introduction of the HFD also prevented disturbances of glucose homeostasis and insulin secretion and did not allow an increase in body weight, which was observed in the group fed only with the HFD. In the latter model, we observed that NAC supplementation resulted in a significant improvement of the salivary mitochondrial function as well as reduced the ADP/ATP ratio compared to HFD rats [5] and strengthened the antioxidative capacity of both glands, but unfortunately protected against oxidative damage only to the parotid glands of IR rats [14].

## 5. Conclusions

1. The inclusion of NAC supplementation at the stage of hyperglycemia effectively improved the general and salivary gland antioxidant status
2. In the presented experimental model, NAC did not eliminate/prevent OS development in the salivary glands
3. Inclusion of NAC supplementation at the hyperglycemia stage only partially prevents salivary gland dysfunction, as manifested by a decrease in stimulated salivary flow. Mechanisms involved in the protein synthesis and secretion react positively to the applied supplementation model, which was observed as increased total protein content in both salivary glands
4. After six weeks of exposure to a high-fat diet, a 4-week NAC supplementation normalized glucose levels, which resulted in normal levels of insulin and HOMA-IR index

**Figure 5:**
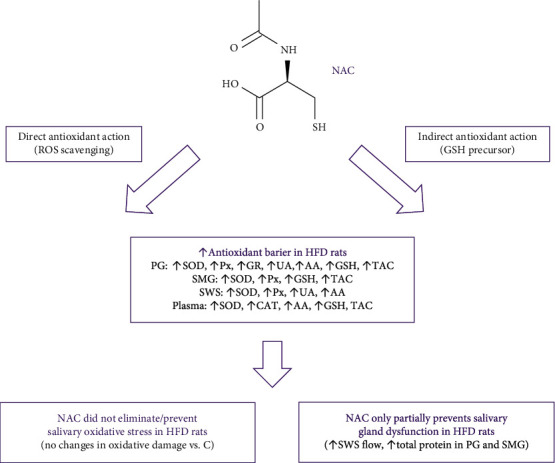
Graphical presentation of conclusions from the research. AA: ascorbic acid; CAT: catalase; NAC: N-acetylcysteine; GR: glutathione reductase; GSH: reduced glutathione; HFD: high-fat diet; Px: salivary peroxidase; PG: parotid glands; SMG: submandibular glands; SOD: superoxide dismutase; SWS: stimulated whole saliva; TAC: total antioxidant capacity; UA: uric acid.

Conclusions from the study are presented in Figure 5.

## Figures and Tables

**Table 1:** General characteristics of rats.

Before NAC supplementation					
	C		HFD		*p* value
Body weight (g)	287 ± 16		349 ± 11		<0.0001
Food intake (mg/day)	17 ± 3.2		12 ± 1.8		<0.0001
Glucose (mg/dL)	96 ± 6.1		157.4 ± 8.9		<0.0001
After NAC supplementation					
	C	C+NAC	HFD	HFD+NAC	ANOVA
Body weight (g)	390.9 ± 10	365.3 ± 12.3	539.3 ± 11.5^a^	438.9 ± 10.4^b^	<0.0001
Food intake (mg/day)	17.47 ± 2.8	15.74 ± 2.2	10.55 ± 2.3^a^	13.64 ± 2.4^b^	<0.0001
Glucose (mg/dL)	97.62 ± 4.3	103.8 ± 10.6	164.8 ± 9.7^a^	121.8 ± 9^b^	<0.0001
Insulin (mIU/mL)	78.65 ± 6.2	80.46 ± 3.5	173.2 ± 12.8^a^	101.8 ± 8.3^b^	<0.0001
HOMA-IR	1.743 ± 0.1	1.828 ± 0.2	6.272 ± 0.7^a^	2.677 ± 0.4^b^	<0.0001

C: control group; HFD: high-fat-fed group; C+NAC: control rats+N-acetylcysteine supplementation; HFD+NAC: high-fat-fed rats+N-acetylcysteine supplementation; HOMA-IR: homeostasis model assessment of insulin resistance; ^a^*p* < 0.05 vs. C; ^b^*p* < 0.05 vs. HFD.

**Table 2:** Salivary gland function of rats.

	C	C+NAC	HFD	HFD+NAC	ANOVA
PG weight (mg)	78.50 ± 4.7	80.89 ± 8.6	104.7 ± 11.6^a^	87.67 ± 4.9^b^	<0.0001
SMG weight (mg)	205.2 ± 15.2	215.4 ± 14.8	261.5 ± 25.7^a^	247.8 ± 22.9	<0.0001
PG index	0.2775 ± 0.02	0.2794 ± 0.04	0.2995 ± 0.04	0.2890 ± 0.04	0.41
SMG index	0.7250 ± 0.07	0.7438 ± 0.08	0.7483 ± 0.08	0.8158 ± 0.09	0.07
TP PG (*μ*g/mL)	3993 ± 291	3953 ± 427	2555 ± 541^a^	4221 ± 495^b^	<0.0001
TP SMG (*μ*g/mL)	3821 ± 363	3695 ± 293	2766 ± 508^a^	3447 ± 592^b^	<0.0001
NWS flow (*μ*L/min)	0.4072 ± 0.07	0.4148 ± 0.09	0.3297 ± 0.05	0.3809 ± 0.09	0.077
SWS flow (*μ*L/min)	90.31 ± 16.3	112.1 ± 24.1	64.67 ± 12.7^a^	71.68 ± 14.8^a^	<0.0001

C: control group; HFD: high-fat-fed group; C+NAC: control rats+N-acetylcysteine supplementation; HFD+NAC: high-fat-fed rats+N-acetylcysteine supplementation; PG: parotid glands; SMG: submandibular glands; TP: total protein; NWS: nonstimulated whole salivary flow; SWS: stimulated whole salivary flow; ^a^*p* < 0.05 vs. C; ^b^*p* < 0.05 vs. HFD.

**Table 3:** Plasma redox status of rats.

	C	C+NAC	HFD	HFD+NAC	ANOVA
Serum					
SOD (mU/mg protein)	0.7602 ± 0.08	0.7191 ± 0.12	0.2952 ± 0.08^a^	0.4730 ± 0.1^b^	<0.0001
CAT (nmol H_2_O_2_/min/mg protein)	1.683 ± 0.08	1.593 ± 0.07	0.7834 ± 0.08^a^	1.117 ± 0.08^b^	<0.0001
GPx (mU/mg protein)	35.16 ± 3.4	33.04 ± 6	35.55 ± 10.7	35.98 ± 8.7	0.2856
GR (mU/mg protein)	10.5 ± 1.6	8.726 ± 2.7	7.789 ± 2.5	9.867 ± 2.5	0.0679
Plasma					
UA (*μ*g/mg protein)	0.3067 ± 0.1	0.3968 ± 0.1	0.4472 ± 0.06^a^	0.3987 ± 0.08	0.0098
AA (pmol/mg protein)	9.592 ± 1.1	10.71 ± 0.7	7.749 ± 1.1^a^	9.084 ± 0.9^b^	<0.0001
GSH (ng/mg protein)	0.7101 ± 0.1	0.7271 ± 0.1	0.2803 ± 0.1^a^	0.6882 ± 0.06^b^	<0.0001
TAC (*μ*mol/mg protein)	0.5865 ± 0.08	0.5971 ± 0.1	0.2798 ± 0.07^a^	0.4405 ± 0.08^b^	<0.0001
TOS (nmol H_2_O_2_ Equiv./mg protein)	1.390 ± 0.4	1.611 ± 0.4	3.789 ± 0.9^a^	3.341 ± 1.2^a^	<0.0001
OSI (TOS/TAC ratio)	2.435 ± 0.9	2.845 ± 0.9	14.11 ± 4.2^a^	8.111 ± 3.9^a,b^	<0.0001
8-isoP (pg/mg protein)	1.046 ± 0.3	0.9416 ± 0.5	2.269 ± 0.8^a^	1.751 ± 0.5^a^	<0.0001
4-HNE (*μ*g/mg protein)	35.33 ± 4.8	33.25 ± 7.9	84.77 ± 12.6^a^	70.71 ± 8.9^a,b^	<0.0001
AGE (AFU/mg protein)	0.8863 ± 0.3	0.7803 ± 0.2	1.704 ± 0.4^a^	1.107 ± 0.3^a^	<0.0001
AOPP (nmol/mg protein)	1.738 ± 0.5	1.781 ± 0.5	2.803 ± 0.6^a^	1.951 ± 0.4^b^	<0.0001
8-OHdG (pg/mg protein)	0.083 ± 0.03	0.102 ± 0.05	0.188 ± 0.06^a^	0.131 ± 0.06	0.0006

C: control group; HFD: high-fat-fed group; C+NAC: control rats+N-acetylcysteine supplementation; HFD+NAC: high-fat-fed rats+N-acetylcysteine supplementation; SOD: superoxide dismutase; CAT: catalase; GPx: glutathione peroxidase; GR: glutathione reductase; UA: uric acid; AA: ascorbic acid; GSH: reduced glutathione; TAC: total antioxidant capacity; TOS: total oxidant status; OSI: oxidative status index; 8-isoP: 8-isoprostanes; 4-HNE: 4 hydroxynonneal protein adducts; AGE: advanced glycation end products; AOPP: advanced oxidation protein products; 8-OHdG: 8-hydroxy-d-guanosine; ^a^*p* < 0.05 vs C; ^b^*p* < 0.05 vs. HFD.

## Data Availability

The article contains complete data used to support the findings of this study.
